# Correlation between the increase in serum uric acid and the rapid decline in kidney function in adults with normal kidney function: a retrospective study in Urumqi, China

**DOI:** 10.1186/s12882-023-03151-z

**Published:** 2023-04-21

**Authors:** Na Li, Xiaoping Yang, Jianrong Wu, Yinghong Wang, Zengliang Wang, Huyati Mu

**Affiliations:** 1 Health Management Center, First Affiliated Hospital of Xinjiang Medical University, Urumqi, China; 2 Department of Cardiology, First Affiliated Hospital of Xinjiang Medical University, Urumqi, China

**Keywords:** Chronic kidney disease, Kidney function, Serum uric acid, Hyperuricemia, The estimated glomerular filtration rate

## Abstract

**Background:**

To examine the association between elevated serum uric acid (SUA) levels and the rapid decline in kidney function by conducting a retrospective cohort study on a physically healthy population in Urumqi, China.

**Methods:**

A cohort study of 2,802 physically healthy people with a normal estimated glomerular filtration rate (eGFR) was investigated from 2018 to 2021. The examination procedure included using questionnaires, taking physical measurements, and blood sampling. The rapid decline in kidney function was defined as eGFR > 5 mL·min^–1^ ·(1.73 m^2^ )^–1^ year. The relationship between elevated SUA levels and the rapid decline in kidney function was assessed.

**Results:**

When performing the three-year retrospective analysis, 688 (28.55%) cases experienced a rapid decline in kidney function, and 52 (1.9%) cases developed chronic kidney disease (CKD). They were divided into the stable group and the rapidly declining kidney function group according to eGFR > 15 mL·min^–1^·(1.73 m^2^ )^–1^. The comparison revealed a greater increase in uric acid in the rapidly declining kidney function group [0.30 (-0.29, 0.82) mg/dL vs. − 0.07(-0.54, 0.37) mg/dL, Z = − 8.822, *P* < 0.001]. The participants were further divided into four groups according to their uric acid levels in 2018 and 2021, which included the normal to normal (N-N) group, the normal to hyperuricemia (HUA) (N-H) group, the HUA to normal (H-N) group, and the persistently HUA (H-H) group. The decrease in eGFR was significantly higher in the N-H group than in the other three groups (χ^2^ = 20.580, *P* < 0.001). The results of the multifactorial logistic regression analysis showed that elevated uric acid was a risk factor for the rapid decline in kidney function (OR = 1.640, *P* < 0.001).

**Conclusion:**

Elevated SUA levels were a risk factor for the rapid decline in kidney function in the Chinese health examination population. Higher SUA levels might predict the occurrence of progressive kidney impairment.

## Background

Chronic kidney disease (CKD) is a severe global health risk. It adversely affects human health and the quality of life, especially in an aging population [1, 2]. Since the 1990s, HUA, hypertension, metabolic syndrome, and other influencing factors have been implicated in CKD [3–5]. An increase in uric acid levels and CKD or new onset CKD were found to be positively correlated. Hence, high uric acid levels might lead to the progression of CKD [6]. Information about the rate of decline in kidney function and its pattern is limited. However, some studies found that accelerated decline in kidney function was associated with high uric acid levels, high blood pressure, and metabolic syndrome [7–9]. Also, rapid deterioration in kidney function is strongly associated with increased complications, cardiovascular events, and all-cause mortality [10]. Uric acid is the end product of purine metabolism in the human body, and it exhibits antioxidant activities within the normal physiological range [11]. In contrast, elevated blood uric acid contributes to the onset of gout, causes insulin resistance, and aggravates cardiovascular diseases [12, 13]. Uric acid levels were positively correlated with a decrease in kidney function in a physically examined population [14, 15]. Based on three years of follow-up, a reduction in the blood uric acid levels was found to delay the decrease in kidney function in elderly patients with hypertension [16]. The prognosis of patients with kidney diseases suffering from HUA was considerably worse than those with normal uric acid levels, and anti-uric acid treatment improved kidney function [17–19]. Elucidating the relationship between blood uric acid levels and changes in kidney function can facilitate enhanced prevention and control of CKD and precise nursing. In this study, we retrospectively analyzed the changes in kidney function and uric acid at baseline and after three years in a cohort of people aged 20 years or older at the health management center of the First Affiliated Hospital of Xinjiang Medical University in Urumqi. We analyzed and assessed the association between blood uric acid levels and changes in kidney function in the examined population. Our findings might provide a theoretical basis for managing CKD.

## Materials and methods

### Population

In this study, the population data of health check-ups in the Health Management Center of the First Affiliated Hospital of Xinjiang Medical University for three consecutive years from January to December 2018 were selected. The inclusion criteria were (1) age ≥ 20 years old and (2) permanent residents of Urumqi (≥ 6 months). The exclusion criteria were (1) patients with incomplete demographic data and clinical data; (2) patients with severe liver or kidney disease and those who were administered drugs that affect blood creatinine and uric acid, and those who underwent any form of kidney replacement therapy; (3) patients with eGFR < 60 mL/min·(1.73 m^2^) [20],

In this study, a stratified and grouped sampling design was used, and the baseline survey was conducted from 2018 to 2019. In 2018 and 2021, a standardized process was adopted for blood sample collection, and 3,472 participants who met the inclusion criteria were included. After the screening process, 121 people were excluded due to the exclusion criteria. An additional 549 participants were excluded because they did not have a health check-up in 2021. In the end, 2,802 participants were eligible to participate in this study, including 1,736 males (61.96%) and 1,066 females (38.04%), who were 20 to 88 years old.

The study was approved by the Ethics Committee of the First Affiliated Hospital of Xinjiang Medical University.

### Data and sample collection

Data collection and other laboratory data: The medical history of the participants, such as their gender, age, ethnicity, medical history, medication, blood pressure, and body mass index (BMI), was recorded using a questionnaire. All participants underwent liver function, lipid, creatinine, uric acid, and fasting blood glucose tests. All participants were fasting (for at least 8 h) during blood collection, and all tests were performed in the laboratory. Serum creatinine and uric acid were measured using an enzymatic method (Olympus AU5811 Roche C800 fully automatic Biochemical Analyzer, Japan).

### Kidney function evaluation

Demographic and clinical data recorded in 2018 were defined as baseline information. Kidney function was evaluated based on the level of eGFR. The levels of eGFR in 2018 and 2021 were defined as kidney function at baseline and endpoint, respectively. The CKD-EPI formula was used to estimate the glomerular filtration rate (eGFR): eGFR = 141 × min (blood creatinine/κ, 1) ɑ × max (blood creatinine/κ, 1)– 1.209 × 0.993Age × 1.018 (if female) (female, κ = 0.7, male κ = 0.9; female ɑ = − 0.329, male ɑ = − 0.411, min refers to Scr/κ min or 1, max refers to Scr/κ max or 1) [21].

### Outcome

The primary outcome was a rapid decrease in eGFR, as defined by a reduction of > 5 mL·min^–1^·(1.73 m^2^ )^–1^/year [22, 23]. A reduction in eGFR of > 15 mL/min·(1.73 m^2^) after three years was considered to indicate the progression of kidney function. The secondary outcome was the development of CKD, as defined by the decrease in the level of eGFR to < 60 mL/min/1.73 m^2^. The participants were divided into a stable kidney function group and a declining kidney function group. HUA was defined by the SUA level, which was ≥ 7 mg/dL in males and ≥ 6 mg/dL in females [24, 25]. The participants were divided into four groups depending on the HUA content in 2018 and 2021, including the normal to normal (N-N) group, the normal to HUA (N-H) group. The HUA reduced to normal (H-N) group and the persistent HUA (H-H) group. The changes in the glomerular filtration rate (∆eGFR = eGFR2021-eGFR2018) and uric acid (∆UA = UA2021-UA2018) in the four groups were analyzed.

### Quality control

The investigators were trained and assessed before starting the study. Various data obtained during the survey were managed on a computer using entry forms designed by epidemiologists. The data were dually recorded using the EpiData 3.1 software to create a database. In 2018 and 2021, a standardized process was used for blood sample collection, Laboratory quality control was also conducted.

### Statistical analysis

The data were expressed as the mean ± standard deviations (SDs) for continuous variables and the number and percentage for classified variables. Two independent two-sample tests, i.e., the t-test or the Mann-Whitney U-test, was performed to compare the stable kidney function group and the declining kidney function group, and Pearson’s χ^2^ test was performed to compare the rates. The data of SUA were divided into four groups and tested by the Kruskal-Wallis H-test. The risk factors for the rapid decline in kidney function were analyzed using a logistic regression model. All data were analyzed using SPSS 26.0. All differences between groups were considered to be statistically significant at *P* < 0.05.

## Results

General data: In total, 2,802 individuals aged 21 to 88 years in Urumqi were included in this study to complete the three-year retrospective analysis. There were 1,736 males (61.96%) and 1,066 females (38.04%). The data recorded in 2018 was considered to be the baseline. The participants were 44.65 ± 14.43 years old and had a mean uric acid level of 5.09 ± 1.32 mg/dL. There were 305 cases of HUA with a uric acid level of 7.39 ± 0.75 mg/dL. The mean rate of glomerular filtration was 105.46 ± 19.63 mL·min^–1^ ·(1.73 m^2^ )^–1^ (Table 1).

**Table 1:** The basic demographic and baseline characteristics of the biochemical indicators of 2,802 cases recorded in 2018

Item	Value
Male [cases (%)]	1736(61.96%)
Age (years)	44.65 ±14.43
BMI (kg/m^2^)	24.39 ±3.65
Systolic blood pressure (mm Hg)	119.83 ±17.33
Diastolic blood pressure (mm Hg)	76.26 ±11.65
Fasting blood glucose (mmol/L)	5.08 ±1.11
Total cholesterol (mmol/L)	4.76 ±0.94
Triglycerides (mmol/L)	1.60 ±1.14
HDL (mmol/L)	1.35 ±0.35
Low-density lipoprotein (mmol/L)	2.90 ±0.78
Glutathione transaminase (U/L)	24.51 ±17.80
Glutathione aminotransferase (U/L)	21.58 ±9.21
Urea nitrogen (mmol/L)	4.97 ±1.95
Blood uric acid (mg/dL)	5.09 ±1.32
HUA(n = 305)	7.39 ±0.75
eGFR_2018_	105.46 ±19.63

Comparison of the basic demographic and biochemical indicators after grouping based on ∆eGFR: During the three-year retrospective analysis, a decrease in the eGFR levels greater than 15 mL·min^–1^ ·(1.73 m^2^)^–1^ was considered to indicate the progression of kidney function. In total, 688 (28.55%) cases experienced a rapid decline in kidney function, and 52 (1.9%) cases developed CKD. There were 688 cases with ∆eGFR > 15 mL·min^–1^ ·(1.73 m^2^)^–1^ in the declining kidney function group and 2,114 cases with ∆eGFR ≤ 15 mL·min^–1^ ·(1.73 m^2^)^–1^ in the stable kidney function group. The basal blood uric acid was not significantly different between the groups, whereas ∆UA was significantly higher in the reduced kidney function group [0.30 (-0.29, 0.82) mg/dL versus − 0.07(–0.54, 0.37) mg/dL, Ζ = − 8.822, *P* < 0.001]. The level of HDL was significantly lower in the reduced kidney function group (1.31 ± 0.34 mmol/L) than in the stable kidney function group (1.37 ± 0.35 mmol/L) (Table 2).

**Table 2:** Comparison of the basic demographic and biochemical indicators between the stable kidney function group and the rapidly declining kidney function group

Item	Stable kidney function group (n = 2114)	Declining kidney function group (n = 688)	*t/Ζ/χ* ^*2*^	*P*
Female (cases)	1312	424	0.042	0.838
Age (years)	44.87 ±14.74	43.98 ±13.43	1.417	0.156
BMI (kg/m^2^)	24.36 ±3.63	24.50 ±3.70	-0.869	0.385
Systolic blood pressure (mm Hg)	119.94 ±17.41	119.50 ±17.06	0.581	0.561
Diastolic blood pressure (mm Hg)	76.23 ±11.65	76.35 ±11.64	-0.234	0.815
Fasting blood glucose (mmol/L)	5.07 ±1.08	5.12 ±1.21	-1.059	0.290
Total cholesterol (mmol/L)	4.76 ±0.96	4.76 ±0.89	0.175	0.861
Triglycerides (mmol/L)	1.59 ±1.17	1.64 ±1.05	-1.054	0.292
HDL (mmol/L)	1.37 ±0.35	1.31 ±0.34	3.920	< 0.001
Low-density lipoprotein (mmol/L)	2.89 ±0.80	2.94 ±0.73	-1.416	0.157
Glutathione transaminase (U/L)	24.25 ±17.91	25.31 ±17.45	-1.355	0.176
Glutathione aminotransferase (U/L)	21.53 ±9.10	21.71 ±9.56	-0.445	0.656
Urea nitrogen (mmol/L)	4.98 ±1.36	4.95 ±3.14	0.287	0.774
UA_2018_ (mg/dL)	5.10 ±1.31	5.04 ±1.36	1.058	0.290
∆UA (mg/dL)	-0.07(-0.54,0.37)	0.30(-0.29,0.82)	-8.822	< 0.001
eGFR_2018_	102.36 ±19.48	114.97 ±16.85	-15.226	< 0.001
∆eGFR	0.83(-7.36,6.72)	-25.34(-29.45,-18.74)	-39.454	< 0.001

**Fig. 1:**
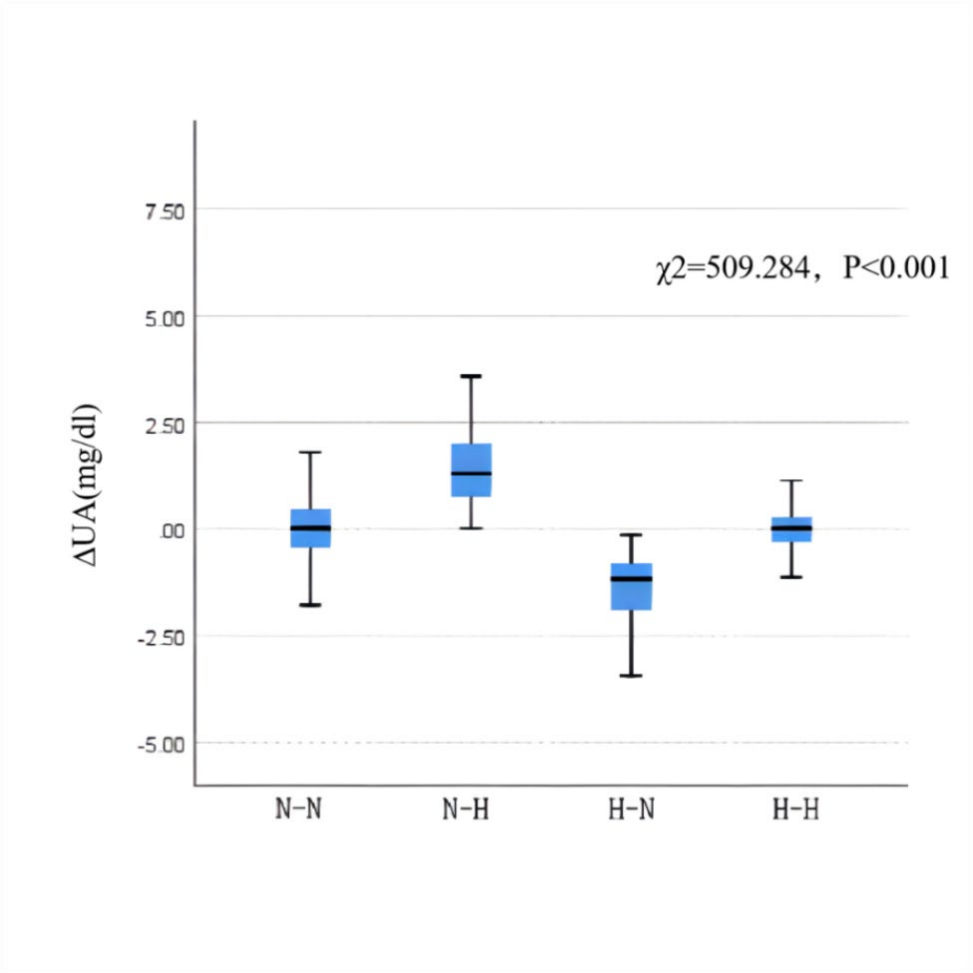
Comparison of ∆eGFR after classification into four groups based on the HUA status N-N: the normal to normal group; N-H: the normal to HUA group; H-N: the HUA to the normal group; H-H: the persistent HUA group; ∆UA is the difference in UA between 2021 and 2018.

**Fig. 2:**
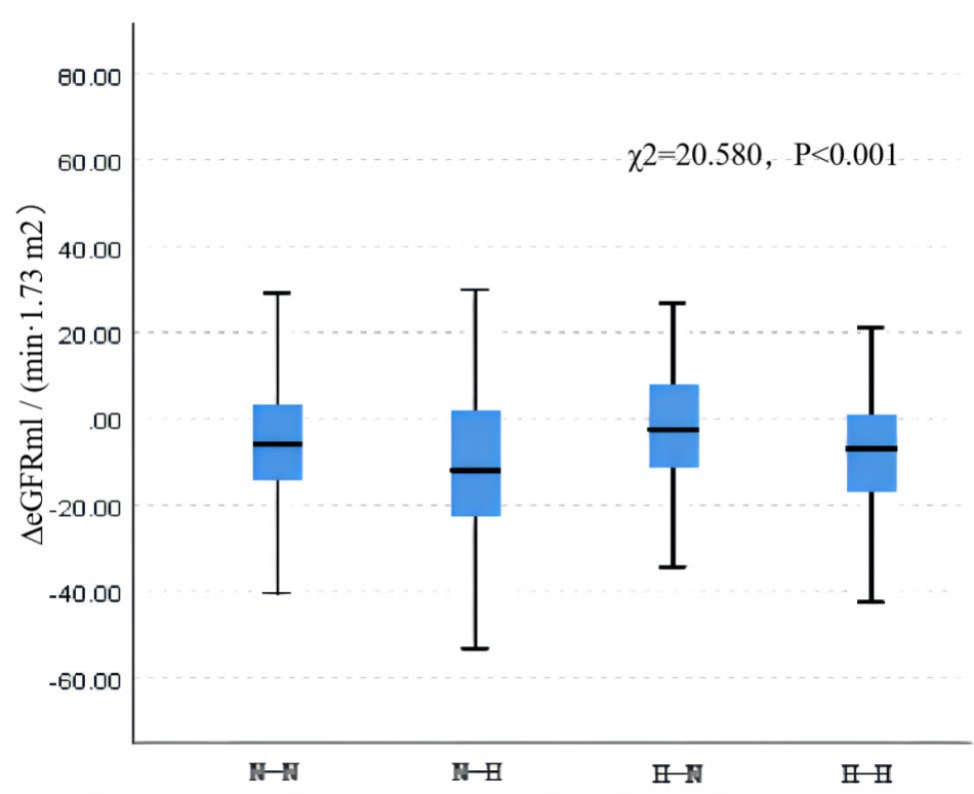
Comparison of ∆UA after classification into four groups based on the HUA status N-N: the normal to normal group; N-H: the normal to HUA group; H-N: the HUA to the normal group; H-H: the persistent HUA group; ∆eGFR is the difference in eGFR between 2021 and 2018.

We also compared ∆eGFR and ∆UA after dividing the participants into four groups based on the status of their HUA content in 2018 and 2021. There were 2,353 cases in the N-N group, 144 cases in the N-H group, 125 cases in the H-N group, and 180 cases in the H-H group. The ∆GFR values were − 5.42 (-14.18, 3.34), − 10.58 (-22.82, 2.21), − 1.69 (-11.68, 8.16), and − 6.63 (-16.99, 1.18) mL·min^–1^ ·(1.73 m^2^ )^–1^, respectively. The level of ∆GFR decreased most significantly in the N-H group (χ^2^ = 20.580, *P* < 0.001) (Fig. 1). The ∆UA was − 0.01 (-0.44, 0.47), 1.51 (0.76, 2.01), − 1.41 (-1.93,–0.80), and 0.00 (-0.30, 0.28) mg/dL in the four groups, respectively, and the differences between groups were statistically significant (χ^2^ = 509.284, *P* < 0.001) (Fig. 2).

### Risk factor analysis for the rapid decline in kidney function

Considering ∆eGFR > − 15 mL·min^–1^ (1.73 m^2^)^–1^ as the endpoint event, a univariate logistic regression analysis was performed to determine the relationship between the influencing factors and the rapid decline in kidney function. The age, gender, BMI, systolic blood pressure, diastolic blood pressure, fasting blood glucose, total cholesterol, triglycerides, HDL, low-density lipoprotein, glutamic aminotransferase, glutamic oxaloacetic aminotransferase, urea nitrogen, basal uric acid values, and changed uric acid values of the individuals were incorporated into the multifactorial logistic regression analysis. An increase in the level of uric acid was considered to be an independent risk factor for the rapid decline in kidney function (OR 1.640, P < 0.001). HDL was considered to be a protective factor for the rapid decline in kidney function (OR 0.685, P = 0.029) (Table 3).

**Table 3:** Multifactorial logistic regression analysis for the rapid decline in kidney function

Influencing factors	Univariate analysis			Multivariate factor analysis		
	Wald *χ*^*2*^	*P*	OR (95% CI)	Wald *χ*^*2*^	*P*	OR (95% CI)
Age (years)	1.281	0.258	0.996(0.988 ~ 1.003)			
HDL(mmol/L)	15.250	< 0.001	0.595(0.458 ~ 0.772)	14.898	0.029	0.685(0.487 ~ 0.962)
LDL(mmol/L)	2.002	0.157	1.082(0.970 ~ 1.207)			
Urea nitrogen (mmol/L)	0.095	0.758	1.007(0.964 ~ 1.052)			
UA_2018_(mg/dL)	0.409	0.522	1.029(0.942 ~ 1.124)			
∆UA(mg/dL)	78.873	< 0.001	1.582(1.429 ~ 1.750)	79.741	< 0.001	1.640(1.471 ~ 1.828)

## Discussion

The global incidence of CKD has increased in recent years. CKD patients suffer from an early and rapid decline in kidney function [26]. HUA is a risk factor for eGFR strata. The lower the eGFR, the higher the prevalence of HUA and gout [27–28]. Hence, early identification of the rapid decline in kidney function during the physical examinations and interventions for the influencing factors is crucial. Among the participants in this study, the increase in blood uric acid was statistically significant in the stable and declining kidney function groups. In contrast, the basal blood uric acid levels showed no statistical significance. Most studies are mainly based on patients with CKD, but studies on cases of normal kidney function with a rapid decline in kidney function are rare. Therefore, in this study, we identified only 688 (28.55%) individuals with a rapid decline in kidney function which further indicated the limitations in assessing early decline in kidney function based on a one-time creatinine value. Thus, routine screening and serial assessment of the glomerular filtration rate are required in the health-screening population to identify patients with rapidly declining kidney function. Early and continual monitoring of the influencing factors and intervention should also be performed. We used the CKD-EPI formula to calculate the glomerular filtration rate [21]. The CKD-EPI formula was developed based on different ethnic groups, mainly comprising CKD patients and healthy individuals. Hence, the applicability of the CKD-EPI formula in the Chinese medical examination population needs to be determined.

The N-H group had the fastest decline in kidney function. Thus, the basal uric acid levels could not predict the rapid decline in kidney function. The elevated level of serum uric acid was an independent risk factor for the rapid decline in kidney function.This finding was similar to that of a study by Cao, where hyperuricemia was a risk factor for kidney impairment in men with normal or mildly impaired kidney function. Even a slight increase in SUA might be a risk factor in people with mild kidney insufficiency [29]. Lowering the blood uric acid levels protects the kidneys by reducing oxidative stress and decreasing the activation of the renin-angiotensin-aldosterone system [23, 30, 31]. Also, when uric acid-lowering drugs, such as allopurinol and febuxostat, are incorporated, the production of inflammatory factors decreases, and endothelial functions improve. This can provide further protection to the kidneys [32, 33]. Some studies found that hyperuricemia has a similar risk of cardiovascular and all-cause mortality whether treated with diuretics or not [34]. However, drugs, as an influencing factor, were not incorporated in this study, and further investigation is necessary.

In this study, the grouping of individuals based on the changes in uric acid levels showed interesting patterns. In the H-H and the H-N groups, the decrease in kidney function and the increase in blood uric acid were lower compared to that in the N-H group, which occurred probably because a decrease in uric acid levels was found after hyperuricemia was detected in some individuals of the H-N group. This finding indicated that with the increase in serum uric acid levels, kidney functions decrease rapidly. The increase in high-density lipoprotein was found to have a protective effect against the rapid decline in kidney function. The reasons might be related to the beneficial antioxidant and anti-inflammatory effects of high-density lipoprotein on the kidneys [35, 36]. It could also be because the stable kidney function group had a healthier lifestyle than the rapidly decreasing kidney function group. These issues need to be further investigated in future studies.Our study had some limitations. First, the population of this study was selected from a single health-screening center, where male patients outnumbered female patients. The differences in the distribution of gender and eGFR levels in the study population were not significant. Thus, the sample in this study represented the Urumqi medical examination population to some extent, and the data were not biased. Second, no “gold standard” data on the maximum glomerular filtration rate (mGFR) were available for this study. However, as the objective of this study was longitudinal changes in kidney function, the application of the eGFR formula did not influence the overall results. Hence, the information on the patients taking medication, particularly the information on hyperuricemic patients under anti-uric acid medication, needs to be determined for improving kidney function in some patients. This aspect should be considered in detail in future studies.

In conclusion, we found that basal blood uric acid levels did not determine a decrease in kidney function. Instead, high blood uric acid was an independent risk factor for a decrease in kidney function. Therefore, the efficient identification and screening of early risk factors are crucial for evaluating the rapid decline in kidney function, timely intervention, and treatment. During health check-ups, changes in kidney function and uric acid levels should be determined as they can provide early warning of CKD and help reduce its incidence.

## Data Availability

The data presented in this study are available on request from the corresponding author.

## List of abbreviations

SUA: Serum uric acid
HUA: Hyperuricemia
eGFR: Estimate the glomerular filtration rate
mGFR: Maximum glomerular filtration rate
CKD: Chronic kidney disease
N-N: Normal to normal group
N-H: Normal to HUA group
H-N: HUA to normal group
H-H: Persistently HUA group
BMI: Body mass index
HDL: High-density lipoprotein
LDL: Low-density lipoprotein
CKD-EPI: Chronic Kidney Disease Epidemiology Collaborative
